# E46K mutant α-synuclein is more degradation resistant and exhibits greater toxic effects than wild-type α-synuclein in *Drosophila* models of Parkinson's disease

**DOI:** 10.1371/journal.pone.0218261

**Published:** 2019-06-26

**Authors:** Ryusuke Sakai, Mari Suzuki, Morio Ueyama, Toshihide Takeuchi, Eiko N. Minakawa, Hideki Hayakawa, Kousuke Baba, Hideki Mochizuki, Yoshitaka Nagai

**Affiliations:** 1 Department of Neurotherapeutics, Osaka University Graduate School of Medicine, Suita, Osaka, Japan; 2 Department of Neurology, Osaka University Graduate School of Medicine, Suita, Osaka, Japan; 3 Diabetic Neuropathy Project, Department of Sensory and Motor Systems, Tokyo Metropolitan Institute of Medical Science, Setagaya, Tokyo, Japan; 4 Department of Degenerative Neurological Diseases, National Institute of Neuroscience, National Center of Neurology and Psychiatry, Kodaira, Tokyo, Japan; Louisiana State University Health Sciences Center, UNITED STATES

## Abstract

Parkinson’s disease (PD) is one of the most common neurodegenerative diseases, which is characterized by progressive motor dysfunction as well as non-motor symptoms. Pathological and genetic studies have demonstrated that α-synuclein (αSyn) plays key roles in the pathogenesis of PD. Although several missense mutations in the αSyn gene have been identified as causes of familial PD, the mechanisms underlying the variance in the clinical phenotypes of familial PD caused by different mutations remain elusive. Here, we established novel *Drosophila* models expressing either wild-type (WT) αSyn or one of five αSyn mutants (A30P, E46K, H50Q, G51D, and A53T) using site-specific transgenesis, which express transgenes at equivalent levels. Expression of either WT or mutant αSyn in the compound eyes by the *GMR-GAL4* driver caused mild rough eye phenotypes with no obvious difference among the mutants. Upon pan-neuronal expression by the *nSyb-GAL4* driver, these αSyn-expressing flies showed a progressive decline in locomotor function. Notably, we found that E46K, H50Q, G51D, and A53T αSyn-expressing flies showed earlier onset of locomotor dysfunction than WT αSyn-expressing flies, suggesting their enhanced toxic effects. Whereas mRNA levels of WT and mutant αSyn were almost equivalent, we found that protein expression levels of E46K αSyn were higher than those of WT αSyn. *In vivo* chase experiments using the drug-inducible *GMR-GeneSwitch* driver demonstrated that degradation of E46K αSyn protein was significantly slower than WT αSyn protein, indicating that the E46K αSyn mutant gains resistance to degradation *in vivo*. We therefore conclude that our novel site-specific transgenic fly models expressing either WT or mutant αSyn are useful to explore the mechanisms by which different αSyn mutants gain toxic functions *in vivo*.

## Introduction

Parkinson’s disease (PD) is one of the most common neurodegenerative diseases, and is characterized by progressive motor dysfunction, such as resting tremor, bradykinesia, and rigidity, as well as non-motor symptoms, including olfactory deficit, autonomic dysfunction, and sleep disturbance. The pathological hallmark of PD is the loss of dopaminergic neurons in the substantia nigra, accompanied by the deposition of intraneuronal inclusions called Lewy bodies (LBs), which is comprised mainly of α-synuclein (αSyn). Although the majority of PD cases are sporadic, about 10% of cases are familial, and both missense and multiplication mutations in the αSyn gene (*SNCA*) were discovered to cause familial PD [1–4]. Moreover, genome-wide association studies identified single-nucleotide polymorphisms (SNPs) in the *SNCA* gene to be major risk factors for sporadic PD [5,6]. Considering these pathological and genetic findings, αSyn is thought to play key roles in the pathogenesis of PD.

Several missense mutations of αSyn that are responsible for familial PD have been identified so far, including A30P, E46K, H50Q, G51D, A53E, and A53T [1,7–11]. However, how these different mutations contribute to the pathogenesis of PD still remains elusive. In previous *in vitro* studies, E46K, H50Q, and A53T αSyn have been shown to have higher aggregation propensity than wild-type (WT) αSyn, whereas A30P and G51D αSyn have lower aggregation propensity [12–16]. On the contrary, *in vivo* studies focusing on the aggregation-resistant tetramer and aggregation-prone monomer forms of αSyn reported that A30P, E46K, H50Q, G51D, and A53T mutations decreased tetramer:monomer ratios in cell culture and mouse brains [17–19]. E46K αSyn, but not A30P or A53T αSyn, was also reported to show enhanced phosphorylation of the Ser-129 residue in human cells, yeast, and mouse brains [20]. Considering the prominent importance of αSyn in the pathogenesis of PD, elucidating the pathomechanisms by which αSyn mutations gain neurotoxicity is indispensable to understand PD pathogenesis. To elucidate the pathological effects of αSyn mutations, we established transgenic *Drosophila* models of PD expressing WT αSyn or αSyn mutants using site-specific transgenesis, by which the transgene is inserted into the same locus of the genome, and thus the transgenes are expected to be expressed at equivalent levels [21,22]. This method enables us to precisely compare the effects of each mutation *in vivo*. Using these transgenic *Drosophila* lines, we showed that the neuronal expression of E46K, H50Q, G51D, and A53T αSyn in flies results in stronger toxic effects than the expression of WT αSyn. We found that the protein expression level of E46K αSyn was higher than that of WT αSyn, despite equivalent mRNA expression levels. Furthermore, we demonstrated through *in vivo* chase experiments that degradation of the E46K αSyn protein was significantly delayed compared with WT αSyn. These results imply that one of the pathological effects of the E46K mutation in PD pathogenesis is conferring resistance to degradation.

## Materials and methods

### Fly stocks

Flies were grown on standard cornmeal medium at 25°C. Human WT or mutant (A30P, E46K, H50Q, G51D, or A53T) αSyn transgenic fly lines were generated using phiC31 integrase-mediated site-specific transgenesis (BestGene Inc., Chino Hills, CA). The pcDNA3.1(+) vector containing each mutant αSyn cDNA was generated by site-directed mutagenesis using pcDNA3.1(+)-human WT αSyn cDNA as the template. Prime STAR Max DNA polymerase (Takara Bio Inc., Kusatsu, Japan) was used for the polymerase chain reaction (PCR) and site-directed mutagenesis. Human WT and each mutant αSyn DNA fragment were amplified by PCR with the primers 5′-ACTAGCGGCCGCATGGATGTATTC-3′ and 5′-ACTTGGTACCTTAGGCTTCAGGTTC-3′, digested with NotI and KpnI, and ligated into the pUAST-attB vector (kindly provided by Dr. Johhanes Bischof [23]). Each transgene was inserted into the attP2 site on chromosome 3 of the host flies (Bloomington Stock Center #8622). Transgenic fly lines bearing *GMR-GAL4* have been described previously [24]. Transgenic fly lines bearing *GMR-GeneSwitch* (#6759), *UAS-hWTαSyn*(R) (#8146, random transgenesis) [25], and *nSyb-GAL4* (#68222) were obtained from the Bloomington Stock Center (Bloomington, IN). Male flies were used in all the experiments.

The sequence of primers for site directed mutagenesis are as follows:

A30P forward: 5′-GGGTGTGGCAGAAGCACCAGGAAAGACAAAAGA-3′

A30P reverse: 5′-TCTTTTGTCTTTCCTGGTGCTTCTGCCACACCC-3′

E46K forward: 5′-GGCTCCAAAACCAAGAAGGGAGTGGTGCATG-3′

E46K reverse: 5′-CATGCACCACTCCCTTCTTGGTTTTGGAGCC-3′

H50Q forward: 5′-GGAGGGAGTGGTGCAGGGTGTGGCAACAG-3′

H50Q reverse: 5′-CTGTTGCCACACCCTGCACCACTCCCTCC-3′

G51D forward: 5′-GGGAGTGGTGCATGATGTGGCAACAGTGG-3′

G51D reverse: 5′-CCACTGTTGCCACATCATGCACCACTCCC-3′

A53T forward: 5′-GTGGTGCATGGTGTGACAACAGTGGCTGAGA-3′

A53T reverse: 5′-TCTCAGCCACTGTTGTCACACCATGCACCAC-3′

### Fly eye imaging

Light microscope observation of fly eyes was performed using a stereoscopic microscope (SZX10, Olympus, Tokyo, Japan) with a digital camera unit (DP21, Olympus). Scanning electron microscopic (SEM) images were taken using an electron microscope (TM1000, Hitachi, Tokyo, Japan). One-day-old male adult flies were used.

### Climbing assay

The climbing assay was performed according to a published protocol [26]. Six to twenty male flies were used for each genotype. Climbing scores were obtained from four to five independent experiments.

### Quantitative RT–PCR

Total RNA was isolated from the heads of 1-day-old flies (five heads per sample). cDNA was synthesized from total RNA using the QuantiTect reverse transcription kit (QIAGEN K.K., Tokyo, Japan) according to the manufacturer’s instructions. Quantitative reverse transcription (RT)-PCR was performed with a CFX96 real-time PCR detection system (Bio-Rad Laboratories, Inc., Hercules, CA) using the SYBR Premix Ex Taq II (Takara Bio Inc., Kusatsu, Japan). Data were analyzed using the standard curve method. The sequences of the forward and reverse primers are as follows:

αSyn forward: 5′-AAAACCAAACAGGGTGTGGC-3′

αSyn reverse: 5′-TGCTCCCTCCACTGTCTTCT-3′

Rpl32 forward: 5′-AGCGCACCAAGCACTTCATCCGCCA-3′

Rpl32 reverse: 5′-GCGCACGTTGTGCACCAGGAACTTC-3′

### Immunoblotting

Fly heads were homogenized in Triton lysis buffer (50 mM Tris–HCl, pH 7.4, 1% Triton X-100, 150 mM NaCl, and 1 mM ethylenediaminetetraacetic acid (EDTA)) containing a protease inhibitor mixture (cOmplete, EDTA-free, Roche Applied Science, Indianapolis, IN) and centrifuged at 15,000 *g* for 20 min at 4°C, and the supernatants were collected as the Triton-soluble fractions. For the preparation of Triton-insoluble fractions, the remaining pellets were washed twice with Triton lysis buffer and lysed in sodium dodecyl sulfate (SDS) buffer (2% SDS, 90 mM Tris–HCl pH 6.8, 20% glycerol). For unfractionated samples, fly heads were homogenized in SDS buffer, centrifuged at 12,000 g for 5 min at 4°C, and the supernatants were collected. The proteins were separated by 5%–20% polyacrylamide gels, transferred to polyvinylidene fluoride (PVDF) membranes (Bio-Rad Laboratories, Hercules, CA) and incubated with phosphate-buffered saline containing 0.4% paraformaldehyde for 30 min at room temperature [27] before blocking with PVDF Blocking Reagent for *Can Get Signal* (TOYOBO Co., Ltd., Osaka, Japan). The antibodies used in this study were as follows: anti-αSyn (clone 42, BD Transduction Laboratories, Franklin Lake, NJ), anti-actin (clone AC40, Sigma-Aldrich, St. Louis, MO) at 1:1,000 dilution, and HRP-conjugated secondary antibodies at 1:10,000 dilution (Jackson ImmunoResearch Laboratory, West Grove, PA). The bands were visualized with ImmunoStar Zeta (Wako Pure Chemical Industries, Osaka, Japan), and images were captured by an Asherman Imager 600 (GE Healthcare Life Science, Pittsburgh, PA). Signal intensities were quantified by densitometry using ImageJ v1.50i software (National Institutes of Health, Bethesda, Maryland).

### Quantification of αSyn protein turnover

To assess αSyn protein turnover, the GeneSwitch conditional expression system was used, as previously described [28]. Briefly, RU486 (mifepristone, Sigma-Aldrich) was dissolved in 100% ethanol, further diluted in water, and then mixed with Instant *Drosophila* medium (Carolina Biological Supply Company, Burlington, NC) at a final concentration of 10 μg/mL. For RU486 treatment, flies were raised on RU486-containing medium from the larval stage until adulthood. After eclosion, the flies were put on standard cornmeal medium for the indicated time periods and fly heads were homogenized in SDS buffer and centrifuged at 12,000 g for 5 min at 4°C. The supernatants were then subjected to immunoblot analysis, as described above.

### Statistical analyses

Data were analyzed using Excel 2007 (Microsoft, Redmond, WA) or R version 3.5.2 (The R Foundation for Statistical Computing, Vienna, Austria). One-way ANOVA followed by the Dunnett’s *post hoc* test was used to analyze differences in climbing scores, αSyn mRNA expression levels, and αSyn protein expression levels. The two-tailed Student *t*-test was used to identify differences in the degradation chase experiments.

## Results

### *Drosophila* models of PD expressing αSyn and its mutants at comparable levels

**Fig 1:**
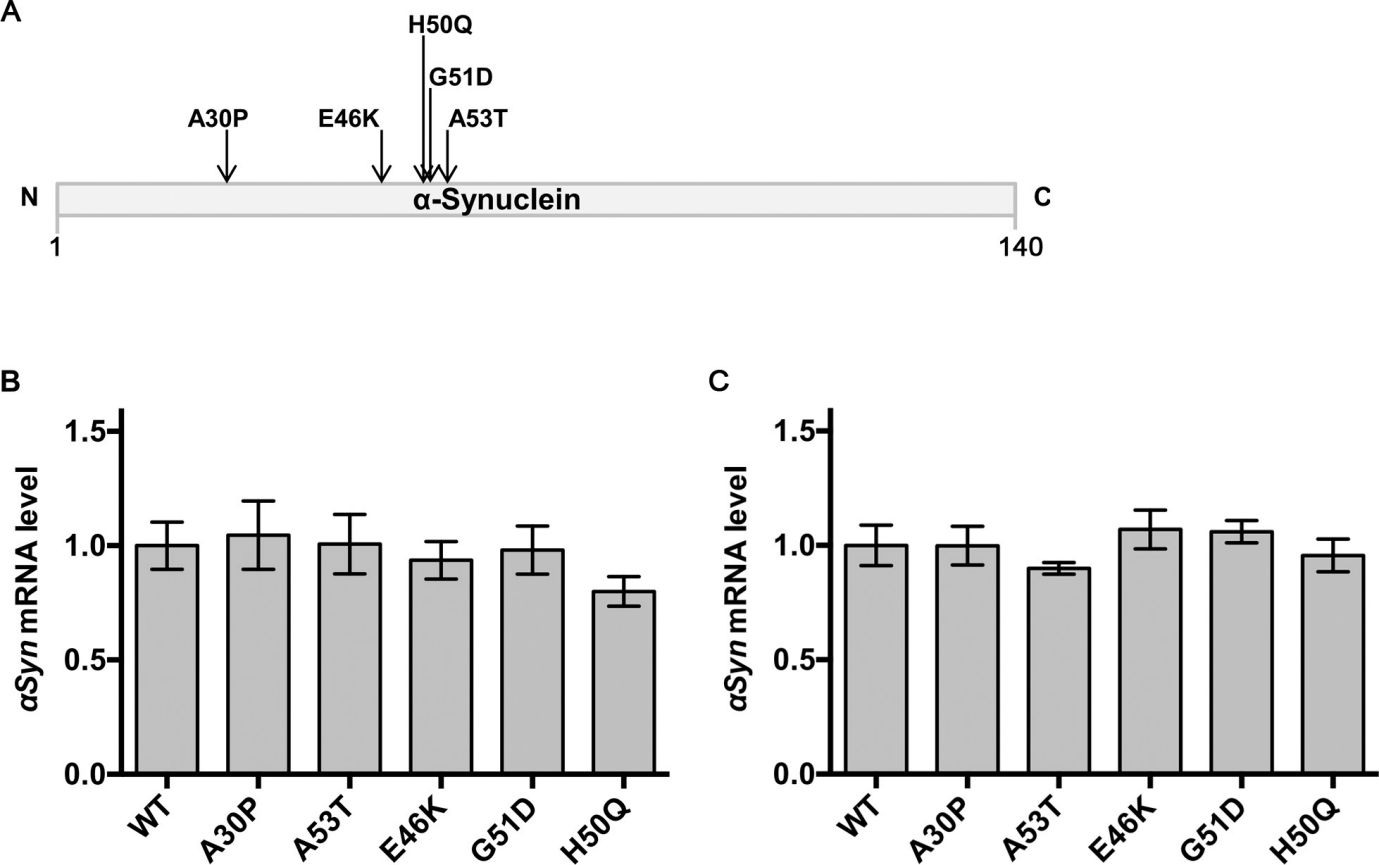
Newly established familial PD model flies express equivalent amounts of WT or mutant αSyn mRNA. (A) Diagram showing αSyn and the locations of the PD-linked mutations used in this study. (B, C) Comparison of relative αSyn mRNA levels demonstrating equivalent expression levels between transgenic flies expressing either WT αSyn or each of the mutants. αSyn was expressed either in compound eyes using the *GMR-GAL4* driver (B) or pan-neuronally using the *nSyb-GAL4* driver (C). Total RNA was obtained from 1-day old male adult fly heads, and used for reverse transcription and quantitative PCR. Each value was normalized to the amount of a ribosomal protein gene *Rpl32* mRNA. The expression level of WT αSyn was set to 1.0. Data are expressed as the mean ± s.e.m. Fly genotypes in (B): WT, *GMR-GAL4*/Y;;*UAS-hWT αSyn*/+; A30P, *GMR-GAL4*/Y;;*UAS-hA30P αSyn*/+; A53T, *GMR-GAL4*/Y;;*UAS-hA53T αSyn*/+; E46K, *GMR-GAL4*/Y;;*UAS-hE46K αSyn*/+; G51D, *GMR-GAL4*/Y;;*UAS*-*hG51D αSyn*/+; H50Q, *GMR-GAL4*/Y;;*UAS-hH50Q αSyn*/+, fly genotypes in (C): WT, +/Y;;*UAS-hWT αSyn*/*nSyb-GAL4*; A30P, +/Y;;*UAS-hA30P αSyn*/*nSyb-GAL4*; A53T, +/Y;;*UAS-hA53T αSyn*/*nSyb-GAL4*; E46K, +/Y;;*UAS-hE46K αSyn*/*nSyb-GAL4*; G51D, +/Y;;*UAS*-*hG51D αSyn*/*nSyb-GAL4*; H50Q, +/Y;;*UAS-hH50Q αSyn*/*nSyb-GAL4*.

To analyze how familial PD mutations affect αSyn toxicity *in vivo*, we generated transgenic *Drosophila* lines expressing WT or mutant forms (A30P, E46K, H50Q, G51D, and A53T) of human αSyn (Fig 1A). Instead of conventional random transgenesis, we employed the site-specific transgenesis system using phiC31 integrase, which mediates sequence-directed integration of transgenes in a distinct genomic locus [21,22]. This method enables us to precisely compare the effects of each mutation, because the expression levels of αSyn are expected to be consistent and position effects of transgenes could be avoided. Indeed, quantitative RT-PCR analysis showed that neither of the mutant αSyn flies showed differences in αSyn mRNA levels compared with WT αSyn flies under the control of the eye-specific *GMR-GAL4* driver or the pan-neuronal *nSyb-GAL4* driver (Figs 1B and 1C). We also confirmed by quantitative RT-PCR that αSyn mRNA levels of all six transgenic flies without the *GAL4* driver were less than 1% of the levels in flies with the *nSyb-GAL4* driver (S1 Fig). This result demonstrates that these newly established transgenic flies produce mRNA transcripts of αSyn at comparable levels. We noted that the αSyn mRNA levels of our flies are 1.5-fold higher than those of the flies previously established by random transgenesis (denoted as WT(R) in S2 Fig) [25].

### Eye-specific expression of WT and mutant forms of αSyn causes comparable compound eye degeneration

**Fig 2:**
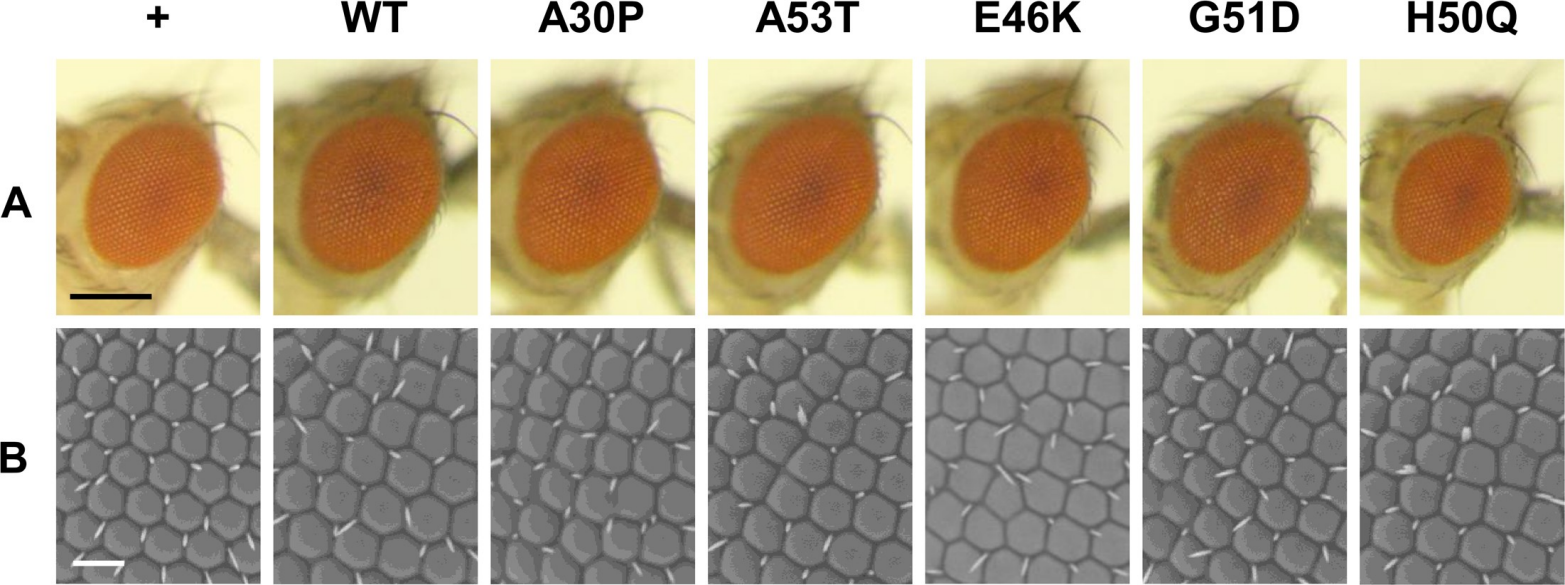
αSyn expression in flies induced mild compound eye degeneration. (A) Light microscopic images of fly eyes showing that there is almost no difference in compound eye morphology among the flies expressing WT and mutant αSyn. Scale bar, 100 μm. (B) SEM images demonstrating eye degeneration, including abnormalities in ommatidial morphology and bristle patterns. However, the extent of degeneration was too mild to detect the difference among the genotypes. Scale bar, 20 μm. Fly genotype: +, *GMR-GAL4*/Y;;*+*/*+*. Other fly genotypes are the same as those in Fig 1B.

It has been reported that the overexpression of WT αSyn in the compound eyes of flies causes the rough eye phenotype as well as abnormal morphology of ommatidia, both of which are readily analyzed by microscopic observation [29–32]. To compare the toxicity of αSyn with various familial mutations, we analyzed the compound eye morphology of flies expressing either WT or mutant αSyn. Light microscopic observation demonstrated that WT αSyn flies showed neither an apparent rough eye phenotype nor loss of pigmentation, similarly to the WT(R) flies (Figs 2A and S3 Fig). On the other hand, electron microscopic observation of the compound eyes of WT αSyn flies showed morphological abnormalities of the ommatidia and abnormal patterns of interommatidial bristles, both of which were not observed in the control flies (Fig 2B). These results demonstrate that eye-specific expression of WT αSyn causes weak degeneration in the compound eyes.

The flies expressing αSyn with familial PD mutations also showed abnormal ommatidial morphology and bristle patterns, but not an apparent rough eye phenotype nor loss of pigmentation (Fig 2A and 2B). These flies showed eye degeneration at almost similar levels, and no apparent differences in the severity of eye degeneration were observed when compared with the WT αSyn flies. These results indicate that αSyn expression in the compound eyes causes weak degeneration, the extent of which would not be significantly affected by familial mutations.

### Neuronal expression of A53T, E46K, G51D, and H50Q αSyn causes an earlier decline in locomotor function than WT αSyn

**Fig 3:**
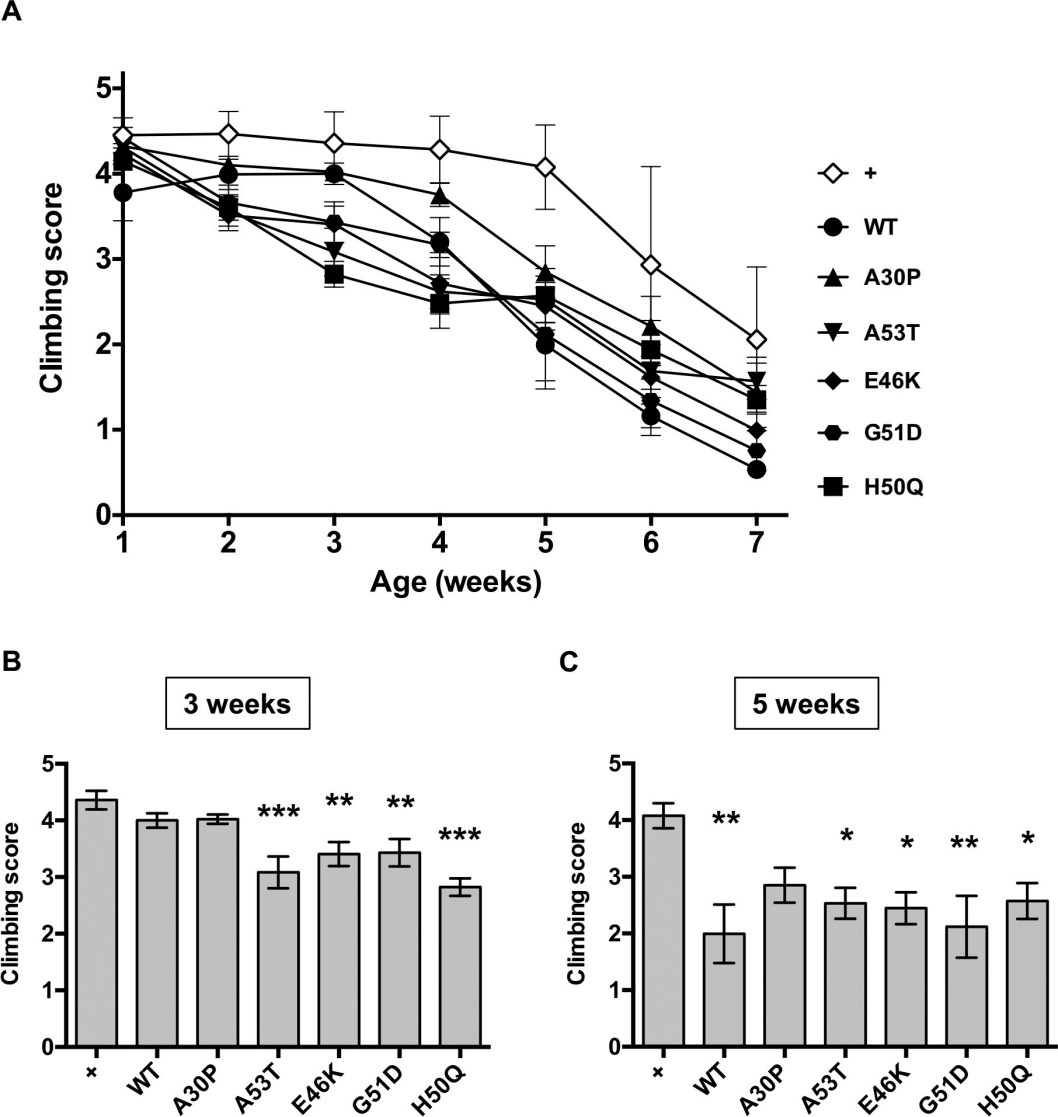
Neuronal expression of A53T, E46K, G51D, and H50Q αSyn showed an early decline in locomotor function. (A) An age-dependent decline in climbing score was observed in flies expressing αSyn in neurons. αSyn expression was induced by the pan-neuronal *nSyb-GAL4* driver. (B) Climbing scores at 3 weeks. Flies expressing either A53T, E46K, G51D, or H50Q αSyn showed a significant decrease in locomotor function compared with control flies, whereas flies expressing WT αSyn did not. (C) Climbing scores at 5 weeks. Flies expressing each of the five forms of αSyn, except for A30P, showed lower climbing scores than control flies. The scores of mutant αSyn-expressing flies were not significantly different from WT αSyn-expressing flies. *P < 0.05, **P < 0.01, and ***P < 0.001 vs control flies (one-way ANOVA followed by the Dunnett’s *post hoc* test). All error bars indicate s.e.m. Fly genotypes are the same as those in Fig 1C. Control fly genotype: +, +/Y;;*nSyb-GAL4*/+.

We next analyzed whether the familial mutations affect the toxicity of αSyn against neuronal functions. It has been reported that neuron-specific expression of WT αSyn in flies causes neuronal dysfunction, which can be detected as the progressive decline of locomotor function [33]. Therefore, we generated flies expressing different forms of αSyn under the control of a pan-neuronal *nSyb-GAL4* driver and analyzed locomotor function by the climbing assay. Quantitative analysis of the climbing scores demonstrated that the locomotor function of WT αSyn flies progressively decreased by 7 weeks of age, which is a much faster decline than that of the control flies bearing *nSyb-GAL4* alone (Fig 3A). This result indicates that *nSyb-GAL4*-mediated expression of WT αSyn causes progressive locomotor dysfunction, which is in good agreement with previous reports in which αSyn was expressed by different neuron-specific drivers, such as *elav-GAL4* [33–36].

Flies expressing mutant αSyn in neurons also showed a progressive decline in locomotor function (Fig 3A). Interestingly, flies expressing either A53T, E46K, G51D, or H50Q αSyn showed a significant decrease in climbing scores compared with control flies from 3 weeks of age, whereas flies expressing WT αSyn did not (Fig 3B). At 5 weeks of age, all mutant αSyn-expressing flies except for A30P αSyn-expressing flies showed decreased locomotor function compared with control flies (Fig 3C), although a significant difference was not detected compared with WT αSyn-expressing flies. These results suggest that WT αSyn and its familial mutants cause locomotor dysfunction upon their neuronal expression, and that several forms of mutations, such as A53T, E46K, G51D, and H50Q enhance αSyn toxicity *in vivo*.

### E46K αSyn mutant accumulates *in vivo*

**Fig 4:**
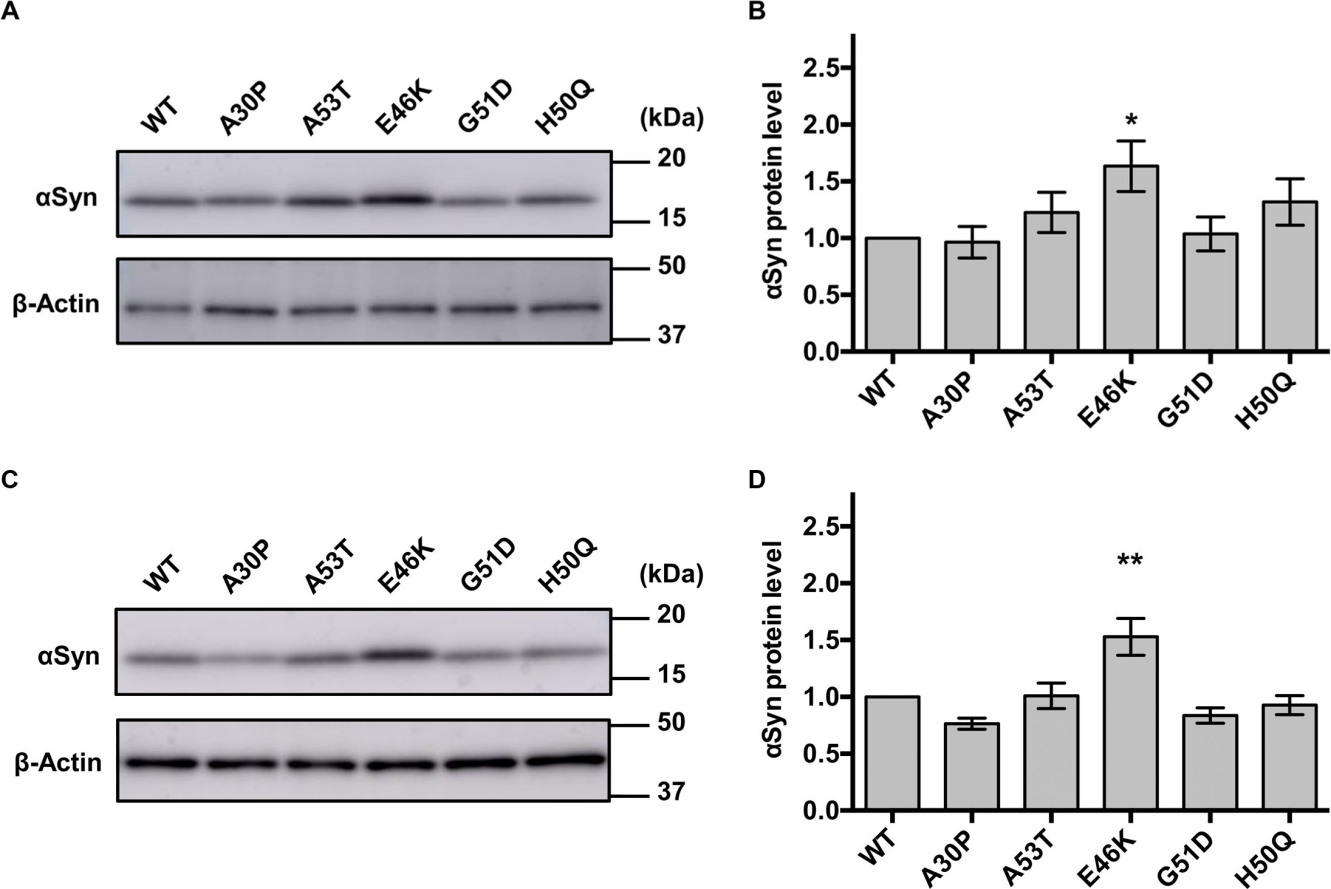
Higher protein levels of E46K αSyn than those of WT αSyn in flies. (A) Triton X-100-soluble fractions were obtained from 1-day-old adult fly heads and subjected to immunoblotting against αSyn. β-Actin was used as a loading control. (B) αSyn protein expression levels were analyzed by densitometry. E46K αSyn showed 1.63-fold higher protein levels than WT αSyn. (C) Total fractions of 1-day-old adult fly heads were subjected to immunoblotting against αSyn. (D) E46K αSyn protein levels were 1.53-fold higher than WT αSyn protein levels. The expression level of WT αSyn was set to 1.0 in B and D. *P < 0.05 and **P < 0.01 compared with WT αSyn flies (one-way ANOVA with Dunnett’s multiple comparisons *post hoc* test). Fly genotypes used in (A) and (B) are the same as those in Fig 1B, and fly genotypes used in (C) and (D) are the same as those in Fig 1C.

Although flies expressing A53T, E46K, G51D, and H50Q αSyn showed an earlier decline in locomotor function, how these mutations accelerate αSyn toxicity remains to be elucidated. Because increased levels of αSyn have been reported to accelerate the onset and progression of disease-associated phenotypes in patients [2–4], we analyzed the protein levels of αSyn in our flies. Immunoblotting analysis of Triton X-100-soluble fractions of eye-specific αSyn-expressing fly homogenates revealed that E46K αSyn-expressing flies showed a 63% increase in αSyn protein level compared with WT αSyn-expressing flies, whereas the other mutant flies showed no differences (Fig 4A and 4B). We noted that αSyn protein levels of our flies are 1.5-fold higher than those of previously established flies (WT(R)) (S4 Fig). Aberrant accumulation and oligomerization of αSyn were not detected in Triton X-100-insoluble fractions (S5 Fig). The analysis of total fractions of pan-neuronally αSyn-expressing fly homogenates also showed a 53% higher αSyn level in E46K αSyn flies than in WTαSyn flies (Figs 4C and 4D). These results suggest that the E46K mutation results in increased monomer protein levels of αSyn, which might be a reason for the accelerated toxicity of this mutation *in vivo*.

### E46K mutation of αSyn gains resistance to degradation *in vivo*

We then analyzed the molecular mechanism underlying how the E46K mutation leads to αSyn accumulation in flies. In general, increased cellular protein levels are caused by increased rates of transcription and translation from each gene, or by delayed rates of protein degradation. Because mRNA levels of the αSyn transgenes are almost consistent among the flies generated in this study (Fig 1B), levels of the αSyn proteins are assumed to be comparable. Therefore, we hypothesized that the E46K mutation might delay αSyn protein degradation, leading to its accumulation *in vivo*.

**Fig 5:**
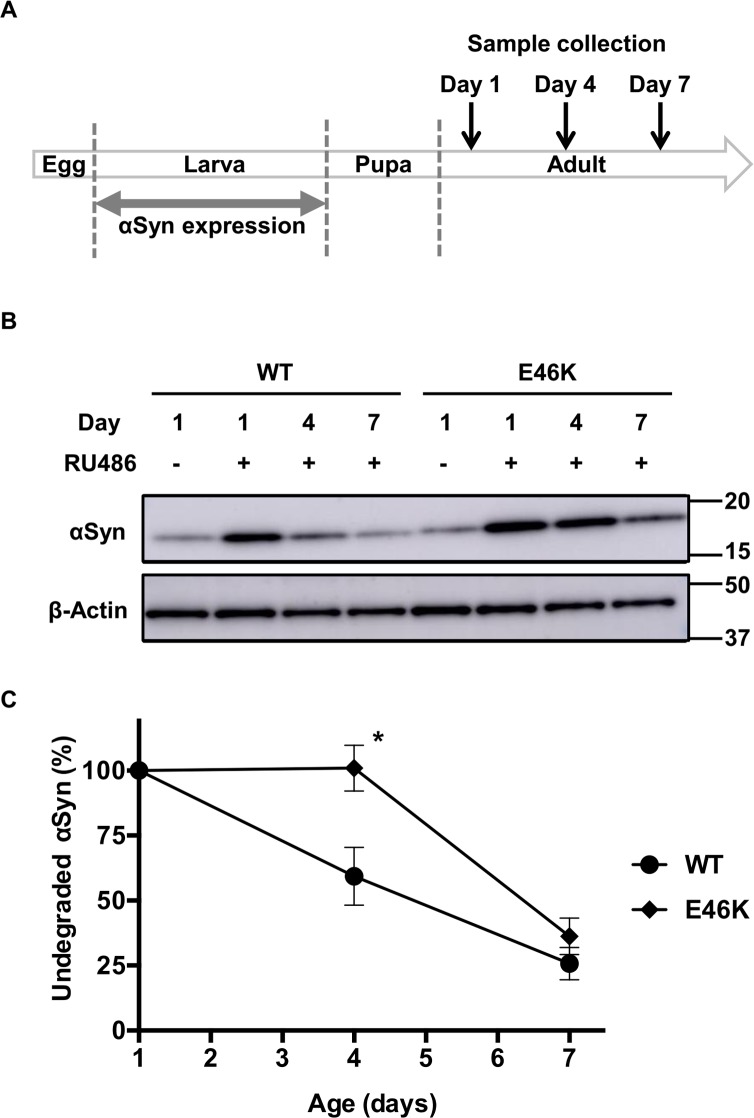
Delayed E46K αSyn degradation compared with WT αSyn degradation. (A) Time course of the conditional expression of αSyn in flies. αSyn expression was transiently induced by the administration of RU486 during the larval stage, and subsequently stopped by withdrawing RU486 during the pupal and adult stages. Adult male flies were collected on day 1, 4, and 7 after eclosion. (B) Immunoblotting analysis of adult fly head lysates using an anti-αSyn antibody. The transient expression of αSyn and time-dependent decrease in αSyn protein levels was observed. (C) The time-dependent protein degradation rate of E46K αSyn was compared with that of WT αSyn. A decrease in undegraded WT αSyn protein levels was observed on day 4, whereas E46K protein levels remained almost unchanged. *P < 0.05 (Student *t*-test). Fly genotypes: WT, *GMR-GeneSwitch*/Y;;*UAS-hWT αSyn*/+; E46K, *GMR-GeneSwitch*/Y;;*UAS-hE46K αSyn*/+.

To test this hypothesis, we employed an inducible expression system using GeneSwitch, in which expression of the genes of interest can be regulated by the addition of mifepristone (RU486) [37]. Using this system, we induced the expression of either WT or E46K αSyn only at the larval stage, and examined the rate of decrease in αSyn protein level after eclosion by immunoblotting analysis of adult flies collected at different time points (Fig 5A). Inducible αSyn expression was confirmed by comparing the amount of αSyn in flies raised in medium containing RU486 with those raised without RU486, although low levels of leak expression were observed (Fig 5B). Interestingly, whereas αSyn protein levels in WT αSyn-expressing flies decreased linearly from eclosion to 7 days of age, levels in E46K αSyn-expressing flies remained at the initial level even at 4 days of age, followed by a decline at 7 days of age (Fig 5C). This result indicates that E46K αSyn shows delayed degradation compared with WT αSyn, which would lead to increased levels of E46K αSyn *in vivo*.

## Discussion

In this study, we established novel *Drosophila* models expressing WT αSyn or αSyn mutants using site-specific transgenesis, which express transgenes at equivalent levels. We showed that flies expressing either E46K, H50Q, G51D, or A53T αSyn show earlier onset of locomotor dysfunction than flies expressing WT αSyn. We found that the expression level of the E46K αSyn protein was higher than that of WT αSyn, despite equivalent mRNA expression levels. *In vivo* chase experiments demonstrated that degradation of the E46K αSyn protein was significantly delayed compared with WT αSyn, indicating that the E46K αSyn has higher resistance to degradation than WT αSyn *in vivo*.

*Drosophila* models of PD expressing not only WT αSyn but also some familial PD-linked forms of mutant αSyn, such as A30P, E46K, H50Q, G51D, and A53T, have been previously established by random transgenesis [25,33,38]. A30P αSyn-expressing flies were reported to demonstrate stronger phenotypes than WT αSyn-expressing flies, although expression levels of the transgenes were not analyzed [33]. Mohite *et al*. also established flies expressing each of E46K, H50Q, and G51D by random transgenesis, and analyzed the fly lines with equivalent protein expression levels [38]. Their E46K, H50Q, and G51D αSyn-expressing flies showed more severe declines in locomotor function than WT αSyn-expressing flies. This is in agreement with our results, implying that these αSyn mutants have increased toxic effects compared with WT αSyn. However, considering that these fly lines were established by random transgenesis [39], they are expected to have different transgene integration sites in the genome, and thus the possibility of position effects cannot be excluded. Therefore, we here used phiC31 integrase-mediated site-specific transgenesis [21] to establish transgenic *Drosophila* lines carrying a single copy of each WT or mutant αSyn transgene in the same genomic locus, which has advantages for directly comparing the effects of different αSyn mutants. In this study, we successfully found a delayed decay of E46K αSyn using our new αSyn transgenic flies established by site-directed transgenesis. Thus, our PD fly models are the first *in vivo* models that are suitable for studying the pathological effects of αSyn mutants.

We examined the effects of αSyn expression in the compound eyes and nervous system of flies. Although αSyn expression in the compound eyes showed mild phenotypes, phenotypic differences among the mutants were not apparent, probably because of the limited sensitivity of quantitative phenotypic evaluation. To quantify the phenotypic differences among the mutants more sensitively, we analyzed their locomotor functions upon neuronal expression of αSyn. E46K, H50Q, G51D, and A53T αSyn-expressing flies demonstrated earlier declines in their climbing scores than WT αSyn-expressing flies. It is noteworthy that familial PD patients carrying E46K, H50Q, G51D, or A53T mutations were reported to develop more severe clinical phenotypes than typical PD, including dementia or cognitive dysfunction [1,8,10], although the number of clinical reports of familial PD patients is limited. A30P αSyn-expressing flies did not show an earlier onset of the climbing phenotype than WT αSyn-expressing flies, which is inconsistent with a previous report [33]. However, since the wild-type and A30P-expressing flies in the previous report were generated by random transgenesis, this discrepancy may be simply owing to differences in protein expression levels and/or position effects of the transgenes. Therefore, severe phenotypes in our E46K, H50Q, G51D, and A53T αSyn-expressing flies may reflect the severe clinical phenotypes of familial PD patients.

Several studies have explored the pathomechanisms of these mutant forms of αSyn responsible for familial PD. Since αSyn aggregates *in vitro*, and accumulates as Lewy bodies in patients’ brains, the aggregation propensity of αSyn mutants has been studied extensively. *In vitro* studies showed higher aggregation propensities of E46K, H50Q, and A53T αSyn than WT αSyn, as shown by thioflavin-T assays [13–16]. On the contrary, G51D αSyn has been reported to have a lower aggregation propensity than WT αSyn [12]. It is also reported that αSyn interacts with membrane lipid components, which could be targets for αSyn toxicity [40–42]. *In vitro* studies showed that A30P and G51D mutations of αSyn had decreased lipid binding, whereas A53T and H50Q mutations did not differ from WT αSyn in their lipid binding [43–46]. Therefore, a universal mechanism of enhanced toxicity that applies to all αSyn mutants still remains elusive, and each mutation may have different and multiple mechanisms for their toxic effects.

In this study, we found that expression levels of the E46K αSyn protein were significantly higher than those of the WT αSyn protein in our fly models, despite their equivalent mRNA levels. We further showed using *in vivo* chase experiments that degradation of the E46K αSyn protein was slower than that of the WT αSyn protein. A previous study using optical pulse-chase experiments reported that the half-life of the Dendra2-tagged E46K αSyn protein did not differ from that of the Dendra2-tagged WT αSyn protein in rat cortical neurons, although possible effects of the Dendra2-tag on αSyn turnover could not completely be excluded [47]. More recently, the E46K αSyn protein was reported to be degraded by both the proteasome and the macroautophagy pathway in PC12 cells, and cycloheximide chase experiments showed that degradation of the E46K αSyn protein was slower than that of the WT αSyn protein, consistent with our results [48], although we did not exclude the possible dysfunction of background degradation systems by expression of E46K αSyn. Considering that the WT αSyn protein was reported to be degraded by both the proteasome and chaperone-mediated autophagy pathway [49][50], and that A30P and A53T αSyn mutants were reported to be resistant to degradation by chaperone-mediated autophagy [50], gaining resistance to degradation may play important roles in the pathogenesis of familial PD. Taking advantage of their suitability for genetic analyses, our PD fly models expressing WT and mutant αSyn at equivalent levels, which were generated by site-specific transgenesis, are useful *in vivo* models to study the pathomechanisms of PD.

## Supporting information

S1 Fig Flies carrying UAS-αSyn transgenes without the *GAL4* driver express almost negligible levels of αSyn mRNA. (A) αSyn mRNA levels of all six transgenic flies without the *GAL4* driver were about 0.8% of the level of WT αSyn flies with the *nSyb-GAL4* driver. (B) PCR products obtained after 28 cycles were separated on 2% agarose gels. αSyn mRNA of all six transgenic flies without the *GAL4* driver was undetectable. Total RNA was obtained from 1-day old male adult fly heads, and was used for reverse transcription and quantitative PCR. Fly genotypes: nSyb/WT, +/Y;;*UAS-hWT αSyn*/*nSyb-GAL4*; +/WT, +/Y;;*UAS-hWT αSyn*/+; +/A30P, +/Y;;*UAS-hA30P αSyn*/+; +/A53T, +/Y;;*UAS-hA53T αSyn*/+; +/E46K, +/Y;;*UAS-hE46K αSyn*/+; +/G51D, +/Y;;*UAS*-*hG51D αSyn*/+; +/H50Q, +/Y;;*UAS-hH50Q αSyn*/+.
(TIF)

S2 Fig Comparison of mRNA levels between the newly established site-directed αSyn transgenic fly line and the random transgenesis αSyn fly line. Relative mRNA expression levels of WT αSyn in the line newly generated by site-specific transgenesis (WT) and the fly line previously established by random transgenesis (WT (R)). The expression level of WT was 1.5-fold higher than that of WT (R). The expression level of WT was set to 1. *P < 0.05 (Student *t*-test) All error bars indicate s.e.m. Fly genotypes: WT, *GMR-GAL4*/Y;;*UAS-hWT αSyn*; WT (R), *GMR-GAL4*/Y;;*UAS-hWT αSyn*(R)/+.
(TIF)

S3 Fig Comparison of eye phenotypes in the newly established site-directed αSyn transgenic fly line with the random transgenesis αSyn fly line. Light microscope and SEM images of the compound eyes of flies expressing WT αSyn from the *GMR-GAL4* driver. Both types of WT αSyn-expressing flies showed mild changes, such as morphological abnormalities in the ommatidia and abnormal patterns of interommatidial bristles detected by SEM (scale bar, 100 μm), although no obvious morphological changes were observed by light microscopy (scale bar, 100 μm). Fly genotypes used are the same as those in S2 Fig.
(TIF)

S4 Fig Comparison of protein expression levels between the newly established site-directed αSyn fly line and the random transgenesis αSyn fly line. Immunobloting analysis of protein expression levels of WT αSyn (WT) in the fly line newly generated by site-specific transgenesis and the previously established fly line (WT (R)) (left). The right panel is a graph of the quantification of the immunobloting results using densitometry. The expression level of WT was set to 1. In addition to mRNA level, αSyn protein expression level of our αSyn fly line was also higher than that of the conventional αSyn fly line. **P < 0.01 (Student *t*-test). All error bars indicate s.e.m. Fly genotypes used are the same as those in S2 Fig.
(TIF)

S5 Fig αSyn was not detected in the insoluble fractions of αSyn-expressing flies. Triton X-100-insoluble fractions were obtained from 1-day-old adult fly heads and subjected to immunoblotting against αSyn. αSyn-positive bands were not detected in Triton X-100-insoluble fractions. WT (soluble) denotes the Triton X-100-soluble fraction of WT αSyn. Fly genotypes are the same as those in Fig 1B.
(TIF)

S1 File Raw data. (XLSX)

## References

[1] Polymeropoulos MH, Lavedan C, Leroy E, Ide SE, Dehejia A, Dutra A, et al Mutation in the α-synuclein gene identified in families with Parkinson’s disease. Science. 1997;276: 2045–2047. 10.1126/science.276.5321.2045 9197268

[2] Singleton AB, Farrer M, Johnson J, Singleton A, Hague S, Kachergus J, et al α-Synuclein locus triplication causes Parkinson’s disease. Science. 2003;302: 841 10.1126/science.1090278 14593171

[3] Chartier-Harlin MC, Kachergus J, Roumier C, Mouroux V, Douay X, Lincoln S, et al α-Synuclein locus duplication as a cause of familial Parkinson’s disease. Lancet. 2004;364: 1167–1169. 10.1016/S0140-6736(04)17103-1 15451224

[4] Ibáñez P, Bonnet AM, Débarges B, Lohmann E, Tison F, Pollak P, et al Causal relation between α-synuclein gene duplication and familial Parkinson’s disease. Lancet. 2004;364: 1169–1171. 10.1016/S0140-6736(04)17104-3 15451225

[5] Satake W, Nakabayashi Y, Mizuta I, Hirota Y, Ito C, Kubo M, et al Genome-wide association study identifies common variants at four loci as genetic risk factors for Parkinson’s disease. Nat Genet. 2009;41: 1303–1307. 10.1038/ng.485 19915576

[6] Simón-Sánchez J, Schulte C, Bras JM, Sharma M, Gibbs JR, Berg D, et al Genome-wide association study reveals genetic risk underlying Parkinson’s disease. Nat Genet. 2009;41: 1308–1312. 10.1038/ng.487 19915575 PMC2787725

[7] Krüger R, Kuhn W, Müller T, Woitalla D, Graeber M, Kösel S, et al Ala30Pro mutation in the gene encoding α-synuclein in Parkinson’s disease. Nat Genet. 1998;18: 106–108. 10.1038/ng0298-106 9462735

[8] Zarranz JJ, Alegre J, Go JC, Lezcano E, Ros R, Ampuero I, et al The new mutation, E46K, of α-synuclein causes Parkinson and Lewy body dementia. Ann Neurol. 2004;55: 164–173. 10.1002/ana.10795 14755719

[9] Lesage S, Anheim M, Letournel F, Bousset L, Pieri L, Madiona K, et al G51D α-synuclein mutation causes a novel Parkinsonian–pyramidal syndrome. Ann Neurol. 2013;73: 459–471. 10.1002/ana.23894 23526723

[10] Appel-Cresswell S, Vilarino-Guell C, Yu I, Shah B, Weir D, Thompson C, et al Alpha-synuclein p.H50Q, a novel pathogenic mutation for Parkinson’s disease. Mov Disord. 2013;28: 811–813. 10.1002/mds.25421 23457019

[11] Pasanen P, Myllykangas L, Siitonen M, Raunio A, Kaakkola S, Lyytinen J, et al A novel α-synuclein mutation A53E associated with atypical multiple system atrophy and Parkinson’s disease-type pathology. Neurobiol Aging. 2014;35: 2180.e1–2180.e5. 10.1016/j.neurobiolaging.2014.03.024 24746362

[12] Fares MB, Ait-Bouziad N, Dikiy I, Mbefo MK, Jovičić A, Kiely A, et al The novel Parkinson’s disease linked mutation G51D attenuates *in vitro* aggregation and membrane binding of α-synuclein, and enhances its secretion and nuclear localization in cells. Hum Mol Genet. 2014;23: 4491–4509. 10.1093/hmg/ddu165 24728187 PMC4119404

[13] Conway KA, Lee SJ, Rochet JC, Ding TT, Williamson RE, Lansbury PT. Acceleration of oligomerization, not fibrillization, is a shared property of both α-synuclein mutations linked to early-onset Parkinson’s disease: Implications for pathogenesis and therapy. Proc Natl Acad Sci USA. 2000;97: 571–576. 10.1073/pnas.97.2.571 10639120 PMC15371

[14] Li J, Uversky VN, Fink AL. Effect of familial Parkinson’s disease point mutations A30P and A53T on the structural properties, aggregation, and fibrillation of human α-synuclein. Biochemistry. 2001;40: 11604–11613. 10.1021/bi010616g 11560511

[15] Ono K, Ikeda T, Takasaki J, Yamada M. Familial Parkinson disease mutations influence α-synuclein assembly. Neurobiol Dis. 2011;43: 715–724. 10.1016/j.nbd.2011.05.025 21684335

[16] Ghosh D, Mondal M, Mohite GM, Singh PK, Ranjan P, Anoop A, et al The Parkinson’s disease-associated H50Q mutation accelerates α-synuclein aggregation *in vitro*. Biochemistry. 2013;52: 6925–6927. 10.1021/bi400999d 24047453

[17] Bartels T, Choi JG, Selkoe DJ. α-Synuclein occurs physiologically as a helically folded tetramer that resists aggregation. Nature. 2011;477: 107–111. 10.1038/nature10324 21841800 PMC3166366

[18] Dettmer U, Newman AJ, Soldner F, Luth ES, Kim NC, von Saucken VE, et al Parkinson-causing α-synuclein missense mutations shift native tetramers to monomers as a mechanism for disease initiation. Nat Commun. 2015;6: 7314 10.1038/ncomms8314 26076669 PMC4490410

[19] Nuber S, Rajsombath M, Minakaki G, Winkler J, Müller CP, Ericsson M, et al Abrogating native α-synuclein tetramers in mice causes a L-DOPA-responsive motor syndrome closely resembling Parkinson’s disease. Neuron. 2018;100: 75–90.e5. 10.1016/j.neuron.2018.09.014 30308173 PMC6211795

[20] Mbefo MK, Fares MB, Paleologou K, Oueslati A, Yin G, Tenreiro S, et al Parkinson disease mutant E46K enhances α-synuclein phosphorylation in mammalian cell lines, in yeast, and *in vivo*. J Biol Chem. 2015;290: 9412–9427. 10.1074/jbc.M114.610774 25657004 PMC4392248

[21] Bateman JR, Lee AM, Wu CT. Site-specific transformation of *Drosophila* via ΦC31 integrase-mediated cassette exchange. Genetics. 2006;173: 769–777. 10.1534/genetics.106.056945 16547094 PMC1526508

[22] Groth AC, Fish M, Nusse R, Calos MP. Construction of transgenic *Drosophila* by using the site-specific integrase from phage phiC31. Genetics. 2004;166: 1775–1782. 10.1534/genetics.166.4.1775 15126397 PMC1470814

[23] Bischof J, Maeda RK, Hediger M, Karch F, Basler K. An optimized transgenesis system for *Drosophila* using germ-line-specific C31 integrases. Proc Natl Acad Sci USA. 2007;104: 3312–3317. 10.1073/pnas.0611511104 17360644 PMC1805588

[24] Yamaguchi M, Hirose F, Inoue YH, Shiraki M, Hayashi Y, Nishi Y, et al Ectopic expression of human p53 inhibits entry into S phase and induces apoptosis in the *Drosophila* eye imaginal disc. Oncogene. 1999;18: 6767–6775. 10.1038/sj.onc.1203113 10597285

[25] Auluck PK, Chan HYE, Trojanowski JQ, Lee VMY, Bonini NM. Chaperone suppression of α-synuclein toxicity in a *Drosophila* model for Parkinson’s disease. Science. 2002;295: 865–868. 10.1126/science.1067389 11823645

[26] Suzuki M, Fujikake N, Takeuchi T, Kohyama-Koganeya A, Nakajima K, Hirabayashi Y, et al Glucocerebrosidase deficiency accelerates the accumulation of proteinase K-resistant α-synuclein and aggravates neurodegeneration in a *Drosophila* model of Parkinson’s disease. Hum Mol Genet. 2015;24: 6675–6686. 10.1093/hmg/ddv372 26362253

[27] Lee BR, Kamitani T. Improved immunodetection of endogenous α-synuclein. PLoS One. 2011;6: e23939 10.1371/journal.pone.0023939 21886844 PMC3158774

[28] Saitoh Y, Fujikake N, Okamoto Y, Popiel HA, Hatanaka Y, Ueyama M, et al P62 plays a protective role in the autophagic degradation of polyglutamine protein oligomers in polyglutamine disease model flies. J Biol Chem. 2015;290: 1442–1453. 10.1074/jbc.M114.590281 25480790 PMC4340391

[29] Yoshida S, Hasegawa T, Suzuki M, Sugeno N, Kobayashi J, Ueyama M, et al Parkinson’s disease-linked *DNAJC13* mutation aggravates alpha-synuclein-induced neurotoxicity through perturbation of endosomal trafficking. Hum Mol Genet. 2018;27: 823–836. 10.1093/hmg/ddy003 29309590

[30] Roy B, Jackson GR. Interactions between tau and α-synuclein augment neurotoxicity in a *Drosophila* model of Parkinson’s disease. Hum Mol Genet. 2014;23: 3008–3023. 10.1093/hmg/ddu011 24430504 PMC4014195

[31] Tue NT, Shimaji K, Tanaka N, Yamaguchi M. Effect of αb-crystallin on protein aggregation in Drosophila. J Biomed Biotechnol. 2012;2012: 252049 10.1155/2012/252049 22505806 PMC3312385

[32] M’Angale PG, Staveley BE. Bcl-2 homologue Debcl enhances α-synuclein -induced phenotypes in Drosophila. PeerJ. 2016;4: e2461 10.7717/peerj.2461 27672511 PMC5028777

[33] Feany MB, Bender WW. A *Drosophila* model of Parkinson’s disease. Nature. 2000;404: 394–398. 10.1038/35006074 10746727

[34] Riemensperger T, Issa A-R, Pech U, Coulom H, Nguyen M-V, Cassar M, et al A single dopamine pathway underlies progressive locomotor deficits in a *Drosophila* model of Parkinson disease. Cell Rep. 2013;5: 952–960. 10.1016/j.celrep.2013.10.032 24239353

[35] Breda C, Nugent ML, Estranero JG, Kyriacou CP, Outeiro TF, Steinert JR, et al Rab11 modulates α-synuclein-mediated defects in synaptic transmission and behaviour. Hum Mol Genet. 2015;24: 1077–1091. 10.1093/hmg/ddu521 25305083 PMC4986550

[36] Ordonez DG, Lee MK, Feany MB. α-Synuclein induces mitochondrial dysfunction through spectrin and the actin cytoskeleton. Neuron. 2018;97: 108–124.e6. 10.1016/j.neuron.2017.11.036 29249285 PMC5755717

[37] Roman G, Davis RL. Conditional expression of UAS-transgenes in the adult eye with a new gene-switch vector system. Genesis. 2002;34: 127–131. 10.1002/gene.10133 12324966

[38] Mohite GM, Dwivedi S, Das S, Kumar R, Paluri S, Mehra S, et al Parkinson’s disease associated α-synuclein familial mutants promote dopaminergic neuronal death in Drosophila melanogaster. ACS Chem Neurosci. 2018;9: 2628–2638. 10.1021/acschemneuro.8b00107 29906099

[39] Rubin GM, Spradling AC. Genetic transformation of *Drosophila* with transposable element vectors. Science. 1982;218: 348–354. 10.1126/science.6289436 6289436

[40] Davidson WS, Jonas A, Clayton DF, George JM. Stabilization of α-synuclein secondary structure upon binding to synthetic membranes. J Biol Chem. 1998;273: 9443–9449. 10.1074/jbc.273.16.9443 9545270

[41] Martinez Z, Zhu M, Han S, Fink AL. GM1 specifically interacts with α-synuclein and inhibits fibrillation. Biochemistry. 2007;46: 1868–1877. 10.1021/bi061749a 17253773

[42] Fantini J, Yahi N. Molecular basis for the glycosphingolipid-binding specificity of α-synuclein: Key role of tyrosine 39 in membrane insertion. J Mol Biol. 2011;408: 654–669. 10.1016/j.jmb.2011.03.009 21396938

[43] Jo E, Fuller N, Rand RP, St George-Hyslop P, Fraser PE. Defective membrane interactions of familial Parkinson’s disease mutant A30P α-synuclein. J Mol Biol. 2002;315: 799–807. 10.1006/jmbi.2001.5269 11812148

[44] Bussell RJ, Eliezer D. Effects of Parkinson’s disease-linked mutations on the structure of lipid-associated α-synuclein. Biochemistry. 2004;43: 4810–4818. 10.1021/bi036135+ 15096050

[45] Khalaf O, Fauvet B, Oueslati A, Dikiy I, Ruggeri FS, Mbefo MK, et al The H50Q mutation enhances α-synuclein aggregation, secretion, and toxicity. J Biol Chem. 2014;289: 21856–21876. 10.1074/jbc.M114.553297 24936070 PMC4139205

[46] Ruf VC, Nübling GS, Willikens S, Shi S, Schmidt F, Levin J, et al Different effects of α-synuclein mutants on lipid binding and aggregation detected by single molecule fluorescence spectroscopy and ThT fluorescence-based measurements. ACS Chem Neurosci. 2019;10: 1649–1659. 10.1021/acschemneuro.8b00579 30605594

[47] Íñigo-Marco I, Valencia M, Larrea L, Bugallo R, Martínez-Goikoetxea M, Zuriguel I, et al E46K α-synuclein pathological mutation causes cell-autonomous toxicity without altering protein turnover or aggregation. Proc Natl Acad Sci USA. 2017;114: E8274–E8283. 10.1073/pnas.1703420114 28900007 PMC5625897

[48] Yan J, Yuan Y, Chu S, Li G, Chen N. E46K mutant α-synuclein is degraded by both proteasome and macroautophagy pathway. Molecules. 2018;23: 2839 10.3390/molecules23112839 30388770 PMC6278282

[49] Webb JL, Ravikumar B, Atkins J, Skepper JN, Rubinsztein DC. α-Synuclein is degraded by both autophagy and the proteasome. J Biol Chem. 2003;278: 25009–25013. 10.1074/jbc.M300227200 12719433

[50] Cuervo AM, Stefanis L, Fredenburg R, Lansbury PT, Sulzer D. Impaired degradation of mutant α-synuclein by chaperone-mediated autophagy. Science. 2004;305: 1292–5. 10.1126/science.1101738 15333840

