# Supplementary figures and images for: E46K mutant α-synuclein is more degradation resistant and exhibits greater toxic effects than wild-type α-synuclein in Drosophila models of Parkinson's disease
Source: PLoS One. 2019 Jun 26;14(6):e0218261. doi: 10.1371/journal.pone.0218261 (PMC6594597; doi:10.1371/journal.pone.0218261)

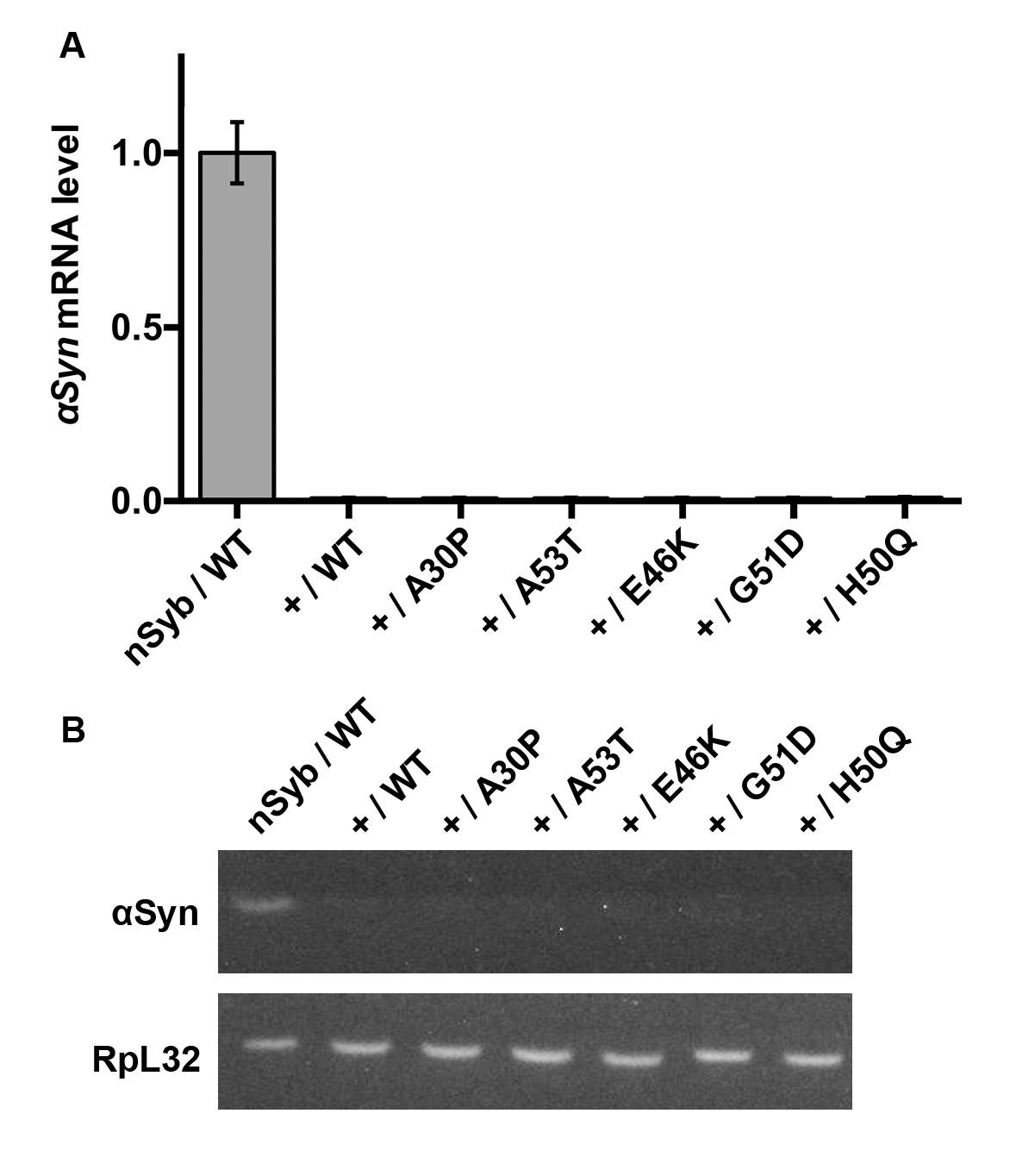

Supplement: S1 Fig — (A) αSyn mRNA levels of all six transgenic flies without the GAL4 driver were about 0.8% of the level of WT αSyn flies with the nSyb-GAL4 driver. (B) PCR products obtained after 28 cycles were separated on 2% agarose gels. αSyn mRNA of all six transgenic flies without the GAL4 driver was undetectable. Total RNA was obtained from 1-day old male adult fly heads, and was used for reverse transcription and quantitative PCR. Fly genotypes: nSyb/WT, +/Y;;UAS-hWT αSyn/nSyb-GAL4; +/WT, +/Y;;UAS-hWT αSyn/+; +/A30P, +/Y;;UAS-hA30P αSyn/+; +/A53T, +/Y;;UAS-hA53T αSyn/+; +/E46K, +/Y;;UAS-hE46K αSyn/+; +/G51D, +/Y;;UAS-hG51D αSyn/+; +/H50Q, +/Y;;UAS-hH50Q αSyn/+. (TIF) [file pone.0218261.s001.tif]

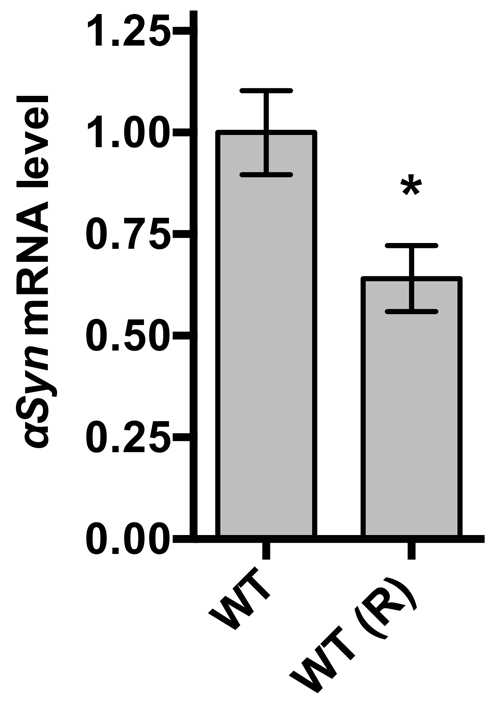

Supplement: S2 Fig — Relative mRNA expression levels of WT αSyn in the line newly generated by site-specific transgenesis (WT) and the fly line previously established by random transgenesis (WT (R)). The expression level of WT was 1.5-fold higher than that of WT (R). The expression level of WT was set to 1. *P < 0.05 (Student t-test) All error bars indicate s.e.m. Fly genotypes: WT, GMR-GAL4/Y;;UAS-hWT αSyn; WT (R), GMR-GAL4/Y;;UAS-hWT αSyn(R)/+. (TIF) [file pone.0218261.s002.tif]

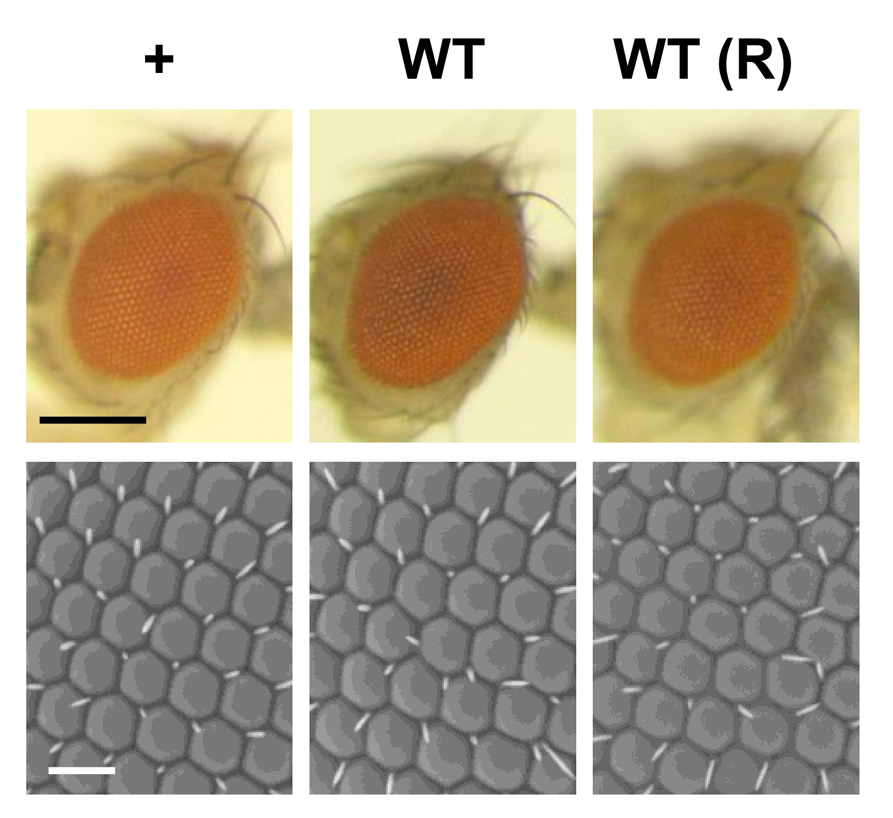

Supplement: S3 Fig — Light microscope and SEM images of the compound eyes of flies expressing WT αSyn from the GMR-GAL4 driver. Both types of WT αSyn-expressing flies showed mild changes, such as morphological abnormalities in the ommatidia and abnormal patterns of interommatidial bristles detected by SEM (scale bar, 100 μm), although no obvious morphological changes were observed by light microscopy (scale bar, 100 μm). Fly genotypes used are the same as those in S2 Fig. (TIF) [file pone.0218261.s003.tif]

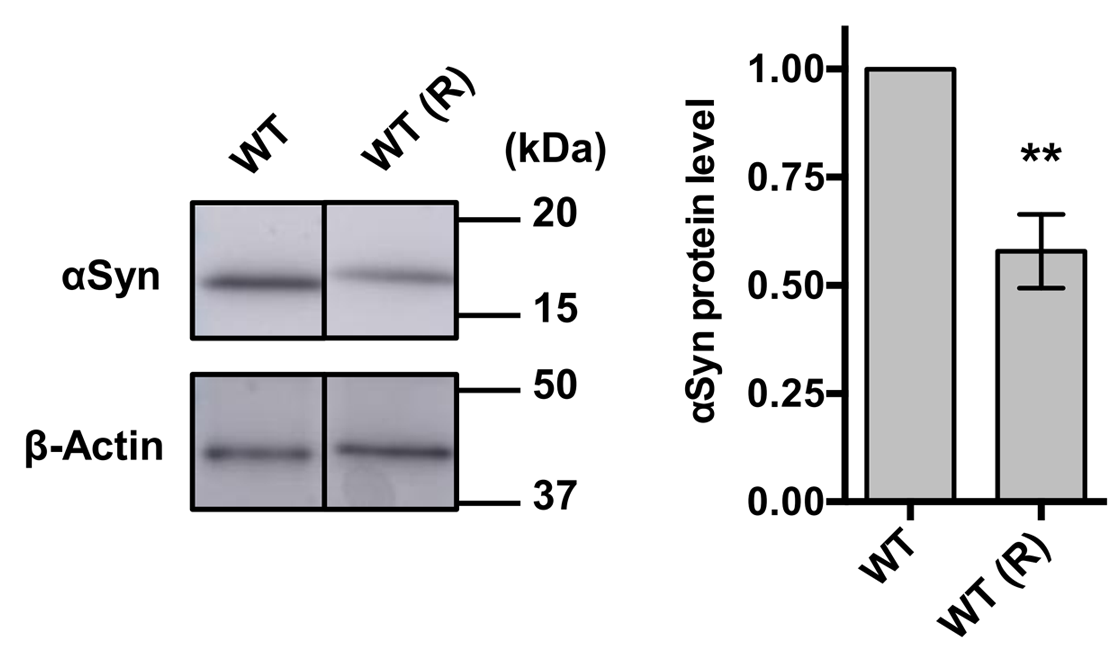

Supplement: S4 Fig — Immunobloting analysis of protein expression levels of WT αSyn (WT) in the fly line newly generated by site-specific transgenesis and the previously established fly line (WT (R)) (left). The right panel is a graph of the quantification of the immunobloting results using densitometry. The expression level of WT was set to 1. In addition to mRNA level, αSyn protein expression level of our αSyn fly line was also higher than that of the conventional αSyn fly line. **P < 0.01 (Student t-test). All error bars indicate s.e.m. Fly genotypes used are the same as those in S2 Fig. (TIF) [file pone.0218261.s004.tif]

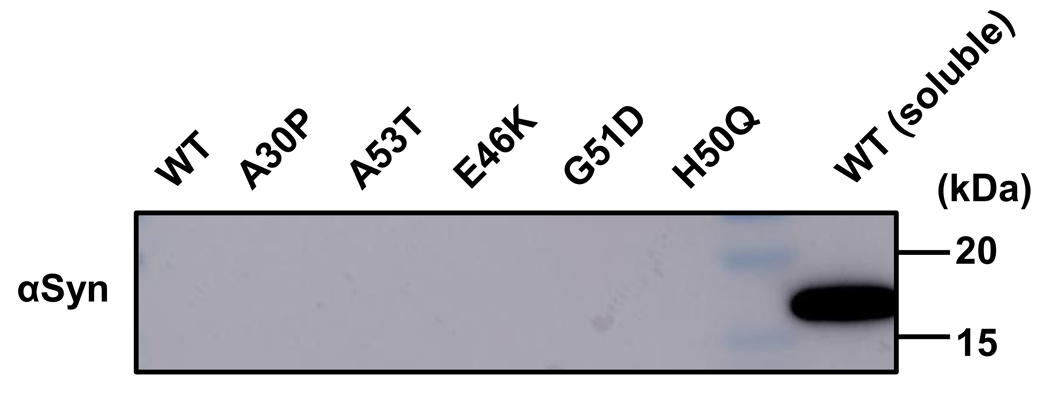

Supplement: S5 Fig — Triton X-100-insoluble fractions were obtained from 1-day-old adult fly heads and subjected to immunoblotting against αSyn. αSyn-positive bands were not detected in Triton X-100-insoluble fractions. WT (soluble) denotes the Triton X-100-soluble fraction of WT αSyn. Fly genotypes are the same as those in Fig 1B. (TIF) [file pone.0218261.s005.tif]
